# Impact of results-based financing on effective obstetric care coverage: evidence from a quasi-experimental study in Malawi

**DOI:** 10.1186/s12913-018-3589-5

**Published:** 2018-10-19

**Authors:** Stephan Brenner, Jacob Mazalale, Danielle Wilhelm, Robin C Nesbitt, Terhi J Lohela, Jobiba Chinkhumba, Julia Lohmann, Adamson S Muula, Manuela De Allegri

**Affiliations:** 1 Heidelberg Institute of Global Health, Ruprecht-Karls Universität Heidelberg, Im Neuenheimer Feld 130.3, 69120 Heidelberg, Germany; 2 Department of Economics, Chancellor College, University of Malawi, Zomba, Malawi; 3 Department of Public Health, University of Helsinki, P.O. Box 20, FI-00014 Helsinki, Finland; 4 College of Medicine, University of Malawi, Blantyre, Malawi; 5 Department of Public Health, School of Public Health and Family Medicine, University of Malawi, Blantyre, Malawi

**Keywords:** Results-based financing, Effective coverage, Maternal and child health, Quality of care, Health care financing

## Abstract

**Background:**

Results-based financing (RBF) describes health system approaches addressing both service quality and use. Effective coverage is a metric measuring progress towards universal health coverage (UHC). Although considered a means towards achieving UHC in settings with weak health financing modalities, the impact of RBF on effective coverage has not been explicitly studied.

**Methods:**

Malawi introduced the *Results-Based Financing For Maternal and Neonatal Health* (RBF4MNH) *Initiative* in 2013 to improve quality of maternal and newborn health services at emergency obstetric care facilities. Using a quasi-experimental design, we examined the impact of the RBF4MNH on both crude and effective coverage of pregnant women across four districts during the two years following implementation.

**Results:**

There was no effect on crude coverage. With a larger proportion of women in intervention areas receiving more effective care over time, the overall net increase in effective coverage was 7.1%-points (*p* = 0.07). The strongest impact on effective coverage (31.0%-point increase, *p* = 0.02) occurred only at lower cut-off level (60% of maximum score) of obstetric care effectiveness. Design-specific and wider health system factors likely limited the program’s potential to produce stronger effects.

**Conclusion:**

The RBF4MNH improved effective coverage of pregnant women and seems to be a promising reform approach towards reaching UHC. Given the short study period, the full potential of the current RBF scheme has likely not yet been reached.

**Electronic supplementary material:**

The online version of this article (10.1186/s12913-018-3589-5) contains supplementary material, which is available to authorized users.

## Background

*Universal health coverage* (UHC) aims to ensure access to needed services of sufficient quality for everyone without suffering financial hardship [1]. Health financing reforms are one means towards reaching this aim in the most efficient and sustainable way. *Results-based financing* (RBF) is one of several health financing reforms currently implemented in a number of low- and middle-income countries (LMIC) [2]. RBF is considered a form of strategic purchasing that reimburses healthcare providers (i.e. service suppliers) based on pre-defined quantity or quality outputs [3]. RBF schemes can also include demand-side components related to population coverage (e.g. vouchers, conditional cash transfers) to further complement service supply [4].

While many LMIC have introduced RBF to improve coverage and quality of maternal and childcare service provision, recent evidence points at the existence of mixed results when evaluating RBF programs, with both quantity and quality indicators responding differently in different contexts [5, 6]. RBF programs are rather complex health financing interventions in terms of their expected theory of change (i.e. use of financial incentives to align health worker behaviours to provide services more efficiently and effectively, thus improving both the quantity and quality of healthcare services used by the targeted population). To reflect this complexity, RBF evaluations often assess impact along a number of different dimensions (i.e. health worker motivation, quality of care, service use, crude population coverage) [7–10]. Given RBF programs’ theoretical impact on both service quality and population coverage, approaches measuring aggregated outcomes that frame broader concepts might be useful adjuncts in gaining additional understanding of the role RBF plays in the achievement of UHC.

To assess achievements towards UHC, *effective coverage* (i.e. the proportion of a population in need of care who receive services of sufficient quality) has been suggested as a suitable measure in the evaluation and monitoring of health system interventions [11]. Effective coverage differs from the commonly used measure of *crude* or *contact coverage* (i.e. the proportion of a population in need of care able to access a service) in also accounting for the expected or actual effectiveness of received care necessary to produce a desired health outcome [12]. While increasingly applied to both general or impact assessments of maternal and child health programs in LMIC [13–15], the use of effective coverage in the evaluation of health financing interventions, such as RBF, has so far not been reported.

Given that the wider focus of RBF programs is not only intervening with health care supply, but also – directly or indirectly – with demand of services and the health system in general [16], the theory of change of RBF closely represents the UHC idea of service coverage (i.e. people in need receive essential health services of sufficient quality) [17]. The aim of this study was therefore to examine the impact of an RBF scheme in Malawi on effective coverage in relation to the provision of facility-based obstetric care services and to gain further insight in the role PBF can play in achieving UHC.

## Methods

### Study setting

At the time the intervention was designed, mortality of mothers in Malawi was high (maternal mortality ratio in 2013: 636 deaths/100,000 live births for Malawi vs. 210 deaths/100,000 live births globally; newborn mortality rate in 2013: 25.9 deaths/1000 live births in Malawi vs. 20.1 deaths/1000 live births globally) [18, 19]. Obstetric care is offered free of charge through public and contracted not-for-profit health facilities [20]. Yet, 75% of all pregnant women with obstetric complications do not actually receive satisfactory emergency obstetric care (EmOC) [21]. Salaries of publicly employed health workers stem directly from central government budgets, while publicly funded primary care facilities (i.e. health centres and district-level hospitals) receive a mix of financial allocations from both central and local government budgets. Malawi’s health system is further challenged by chronic health worker shortages and system-wide stock-outs of essential drugs and supplies [22].

### Intervention

The Malawi Ministry of Health (MoH) introduced the *Results-Based Financing For Maternal And Neonatal Health* (RBF4MNH) Initiative to four districts (Balaka, Dedza, Mchinji, Ntcheu) in April 2013 to enhance obstetric care provision at facilities designated to eventually fully function as EmOC centres. Together, these four districts consist of a total of 33 designated EmOC facilities serving a catchment population with an expected birth rate of about 111,450 deliveries per year [21]. District selection was driven by MoH decisions to avoid overlap with other existing or upcoming maternal health or health financing programs in the country. The RBF4MNH’s main objective is to improve both quality and access to facility-based obstetric care for women and newborns during birth and up to 48 h after delivery [23] through a combination of supply- (performance-based payments to facilities and district health management teams (DHMT)) and demand-side mechanisms (conditional cash transfers (CCT) to pregnant women within catchment areas). Implementation occurred in two phases: an early pilot phase (April 2013 to October 2014) and a later expansion phase (November 2014 onwards). During the early phase, only 18 out of the 33 EmOC facilities (four hospitals, 14 health centres) were empanelled and later expanded by an additional 10 facilities (two community hospitals, eight health centres) with on-going plans for a nation-wide scale-up. Empaneled facilities were selected based on the presence of at least four skilled birth attendants, catchment population size, and number of institutional deliveries.

Upon verification, facilities receive payments for achieving a set of performance targets related to quality of clinical care as well as general service improvement performance indicators [see Additional file 1]. Of these rewards, 40% are earmarked for further investments improving structural working conditions, 60% for individual bonus payments to health workers and auxiliary staff (about 15–25% of staff’s baseline salary). DHMT receive payments related to performance indicators on effective management and support of the entire district [see Additional file 1), also divided into an investment and bonus portion. CCT portions to women were calculated to defray upfront costs related to childbirth (i.e. transportation, basic childbirth items, stay in maternal waiting home) and average opportunity costs anticipated by an average poor patient or her family in Malawi during a 48-h postpartum facility stay. The maximum payment per woman is about seven Euros. To ensure minimum standards in EmOC delivery, all RBF facilities received some basic infrastructure and/or equipment support (e.g. delivery beds, essential examination, EmOC and sterilization material, renovation or reconstruction of labour rooms, postpartum wards, maternal waiting homes, electricity and water supply) prior to intervention launch.

The early implementation phase consisted of three six-month reward cycles and served as an opportunity for implementers to further fine-tune the intervention in response to unforeseen challenges. Feedback and experiences gained during these initial cycles resulted in some programmatic adjustments prior to its expansion in October 2014, including:

- Performance verification using reciprocal peer-review between districts assigned to an external third party auditor to avoid negative sentiments between peers.
- ‘Target-based’ (i.e. all-or-nothing) calculation of rewards changed to ‘proportion-based’ (i.e. relative to progress) to increase motivation towards attainment of more challenging targets.
- Reward cycles reduced from six to three months to increase motivation linking performance and rewards over shorter time intervals.
- With the demand-side CCT component requiring facilities to directly keep and manage cash, administrative restrictions in setting up bank accounts at RBF facilities had to be overcome. This delayed the initial start of the demand-side component to September 2013 (i.e. six months after the initially planned launch).

### Study design

As part of a larger impact evaluation assessing the effect of the RBF4MNH on MNH service utilization and quality [24], this study followed a non-randomized controlled pre-post-test design including 32 of the 33 facilities targeted by the RBF4MNH intervention (we excluded one facility since it could not be identified as EmOC given lack of a delivery ward at baseline). The 18 facilities empanelled during the early phase served as ‘interventions’. Of the remaining 14 control facilities, five turned into RBF facilities at the start of the expansion phase and were treated as ‘switchers’. Data were collected at three time points: baseline (April–May 2013, before official program launch), midterm (June–July 2014, approximately one year after program launch) and endline (June–July 2015, approximately two years after program launch).

### Study samples and data collection

We used three different samples: a facility sample, a service user sample, and an obstetric case sample. During each survey round, we collected three different sets of data: a facility inventory, direct case observations, and a household-level survey.

The *facility sample* included 32 facilities. During baseline and midterm this sample consisted of 18 intervention and 14 control facilities, and 25 interventions and nine controls during endline (after expansion in October 2014). Selection of interventions followed the RBF empanelment criteria. Controls included all EmOC facilities initially not included by the RBF. *Facility inventories* consisted of a structured checklist collecting information on availability of operational equipment, medicines, and supplies essential to routine and basic emergency obstetric care.

The *service user sample* included 5509 randomly selected women living in catchment areas of sampled facilities who completed a pregnancy within the twelve months preceding each survey date. Women reporting pregnancies that resulted in foetal loss or demise before the third trimester were excluded. A two-stage cluster approach was used to sample eligible women. Structured *household-level questionnaires* collected information on women’s demographic characteristics, health-seeking behaviour during pregnancy, obstetric care service use at birth, birth outcome, and household-specific socio-economic.

The *obstetric case sample* included a total of 383 labouring women who presented to the sampled facilities during the three data collection rounds. Convenience sampling was used to include only cases without obstetric complications to ensure comparability between cases. C*ase observations* consisted of a structured checklist collecting information on birth attendants’ adherence to clinical standard guidelines during routine case management. Content was based on performance standards developed for the Malawi *Performance Quality Improvement* program [25]. Observations started once a labouring woman was admitted to the maternity unit and lasted up to two hours after delivery.

### Outcome variables

We used effective coverage of pregnant women with facility-based basic obstetric care services as main outcome variables and defined it according to the literature [11, 12, 26] as *EC*_*FBD preg*_ = *U*_*FBD preg*_ ∣ *N*_*FBD preg*_ ∗ *Q*_*FBD preg*_ representing effective coverage *(EC)* of pregnant women (*preg*) using facility-based delivery services (*FBD*) at a designated EmOC facility *(U)* providing a given level of quality *(Q)*. Here, *U*_*FBD preg*_ ∣ *N*_*FBD preg*_ denotes FBD service use conditional on true need for basic EmOC (i.e. crude or contact coverage) and defined it as any woman carrying a pregnancy beyond the second trimester [27, 28]. We further defined service use *U*_*FBD preg*_ as any pregnant women who used services at any of the designated EmOC facilities included in our sample (vs. non-facility-based care or facility-based care at a non-EmOC facilities). To determine the expected quality of care received by a woman using FBD services at a given facility, we created a composite score using a content-of-care approach measuring the extent to which obstetric care was provided in adherence with pertinent standards of care based on a combination of input and process indicators taken from the inventories and case observations. In developing the composite score, we followed a standard approach including weighting, aggregation and uncertainty analysis [29]. A detailed outline of this approach and the underlying indicator mapping is provided in the additional files [see Additional file 1 and Additional file 2].

The resulting composite score ranged from 0 (not meeting any of the measured obstetric care standards) to 1 (meeting all standards). In the entire sample, none of the studied facilities actually attained a score of 1 (measured scores were nearly normally distributed with a range from 0.22 to 0.86, median of 0.56 and mean of 0.55), dichotomous categorization of facilities into ‘effective’ (i.e. a score of 1) and ‘less than effective’ (i.e. a score less than 1) was not practical to our analysis. In addition to only measuring effective coverage as the percentage of service use adjusted by the respective quality score, we further created additional binary variables based on different cut-off values within the upper range of the measured scores to assess facilities’ relative achievements towards these sub-levels. These cut-offs were set at scores of 0.5, 0.6, 0.7, and 0.8 representing 50%, 60%, 70%, and 80% of full obstetric care effectiveness. Quality scores were then assigned to each sampled woman based on reported facility use during the previous year, assuming short-term changes in service quality or effectiveness at a given facility to be minimal. Due to missing data for some facilities, we were not able to determine a quality score for each facility during baseline and midterm and consequently could not assign quality information to 141 women, reducing the actual sample available for analysis to 5368 women.

### Analytical approach

We use descriptive statistics and two-sample t-test to compare differences in key characteristics between interventions and controls for each sample. We used frequencies illustrating the distribution of facilities and users by quality score categories over time. To estimated the RBF4MNH impact on crude and effective coverage we used linear regression in a difference-in-differences comparison [30]:

$$ {Y}_i={\beta}_0+{\beta}_1t{1}_i+{\beta}_2{T}_i+{\beta}_3t{1}_i\ast {T}_i+{\beta}_4t{2}_i+{\beta}_5t{2}_i\ast {T}_i+{\beta}_k{X}_{ki}+{\varepsilon}_{it}, $$

with *Y*_*i*_ representing the outcome (crude or effective coverage), *t* the time point (*t1* = midterm, *t2* = endline), *T* the treatment group, and T*t the interaction between treatment and time point (T1*t1 interaction at midterm, T*t2 at endline). Coefficients *β*_*3*_ and *β*_*5*_ represent the effect estimates at midterm and endline, respectively.

Models were further adjusted for potential confounders (denoted by *β*_*k*_*X*_*ki*_): household characteristics (district location, distance to nearest EmOC, socioeconomic status) when modelling effects on both crude and effective coverage; additional facility characteristics (type, ownership) when modelling effects on effective coverage. Household socio-economic status was measured as a relative wealth index based on assets and living conditions using principal component analysis and described in detail elsewhere [31]. We also adjusted for clustering at catchment area level and for the late phase expansion with five initial control facilities switching treatment arms. Given the relatively small number of catchment area clusters we used bootstrapping to improve the accuracy of standard errors for our effect estimates. Given the limitations of our clustered study design, we were only able to detect effect sizes of 0.25 or larger at a significance level of 5%. STATA version 14.1 was used for all statistical analyses.

## Results

### Sample characteristics

Table 1 summarizes distribution and characteristics for each of the three samples included in the analysis. Complete information for both facility inventories and corresponding case observations was available for only 26 facilities at baseline, and for 30 and 32 facilities at midterm and endline. Across time points, proportions of health centres and faith-based facilities were higher in the control arm reflecting the RBF4MNH’s mandate to contract primarily public facilities, including the four district hospitals. For the remaining 5368 women sample sizes differed greatly between study arms reflecting the oversampling of district hospital catchment areas in the intervention group. Statistically significant differences in two-sample t-tests between group means existed for household socioeconomic status (*p* = 0.01 at baseline), with women in the intervention arm on average residing in poorer households. Between 88.6 and 97.0% of women depending on study arm and time point reported facility-based service use, while delivery at any or at catchment area specific EmOC facilities was much lower.

**Table 1:** Distributions and characteristics of sampled facilities, observed cases, and surveyed women

	Study arm	Baseline		Midterm		Endline	
Sampled facilities							
Sample sizes, *n (%)*	*Intervention*	17 (100)		18 (100)		23 (100)	
	*Control*	9 (100)		12 (100)		9 (100)	
Proportion of health centres (i.e. not hospitals), *n (%)*	*Intervention*	13 *(76)*		14 *(78)*		18 *(78)*	
	*Control*	8 *(98)*		11 *(92)*		9 *(100)*	
Proportion of public facilities (i.e. not private non-profit), *n (%)*	*Intervention*	16 *(94)*		17 *(94)*		19 *(83)*	
	*Control*	6 *(67)*		6 *(50)*		6 *(67)*	
Sampled obstetric cases							
Sample sizes, *n (%)*	*Intervention*	61 (100)		106 (100)		131 (100)	
	*Control*	19 (100)		51 (100)		18 (100)	
Observed at health centres, *n (%)*	*Intervention*	27 *(44)*		51 *(48)*		69 *(47)*	
	*Control*	8 *(89)*		45 *(88)*		18 *(100)*	
Observed at public facilities, *n (%)*	*Intervention*	57 *(93)*		103 *(97)*		116 *(89)*	
	*Control*	12 *(63)*		21 *(41)*		11 *(61)*	
Sampled women			p-value*		p-value*		p-value*
Sample sizes, *n (%)*	*Intervention*	1084 (100)		1141 (100)		1380 (100)	
	*Control*	628 (100)		695 (100)		440 (100)	
Women’s age in years, *mean (SD)*	*Intervention*	25.5 (6.3)		25.1 (6.1)		25.2 (6.4)	
	*Control*	25.8 (6.4)	*0.41*	25.0 (5.9)	*0.64*	25.5 (6.3)	*0.35*
Parity in number of births, *mean (SD)*	*Intervention*	3.3 (2.2)		2.9 (1.9)		3.0 (2.0)	
	*Control*	3.3 (2.1)	*0.58*	3.0 (1.9)	*0.87*	3.0 (1.9)	*0.94*
Distance in km to catchment EmOC facility, *mean (SD)*	*Intervention*	5.8 (3.4)		5.7 (3.7)		5.5 (3.5)	
	*Control*	5.6 (3.3)	*0.28*	5.5 (3.0)	*0.32*	5.5 (3.2)	*0.96*
Household SES by wealth quintile^a^, *mean (SD)*	*Intervention*	2.9 (1.4)		2.9 (1.5)		2.9 (1.4)	
	*Control*	3.1 (1.4)	*0.01*	3.1 (1.3)	*0.10*	3.2 (1.4)	*< 0.01*
Service use at any facility including non-EmOC^b^, *% (95-CI)*	*Intervention*	90.6 (86.3—93.6)		94.1 (91.8—95.8)		94.2 (91.9—95.9)	
	*Control*	88.6 (81.6—93.2)	*0.53*	97.0 (94.7—98.3)	*0.03*	96.8 (93.7—98.4)	*0.05*
Service use at an EmOC facility in study area^b^, *% (95-CI)*	*Intervention*	75.4 (65.7—83.1)		79.2 (69.9—86.2)		78.7 (69.9—85.5)	
	*Control*	66.2 (47.0—81.3)	*0.32*	73.8 (56.6—85.9)	*0.49*	64.0 (41.5—81.7)	*0.14*
Service use at catchment EmOC facility ^b^, *% (95-CI)*	*Intervention*	60.3 (46.8—72.4)		65.2 (52.8—75.9)		65.4 (54.2—75.2)	
	*Control*	40.6 (24.3—59.3)	*.07*	45.0 (28.5—62.8)	*.05*	38.5 (22.7—57.2)	*.01*

*EmOC = emergency obstetric care, 95-CI = 95%-confidence interval, n = total number, SD = standard deviations, SES = socio-economic status;*

^a^ *quintile 1 = least wealthy, quintile 5 = most wealthy*

^b^ *confidence intervals adjusted for clustered sampling at catchment area level*

*p-values based on two-sample t-test

### Obstetric quality scores

As shown in Table 2, facilities scored on average relatively low when assessed against the standards included in our composite. While we observed a continuous increase over time in intervention facilities (from an average score of 0.55 to 0.65), scores in control facilities showed an increase only at midterm (from 0.56 to 0.60), but then dropped below the baseline value at endline. As for the different score levels, more than half of facilities in each study arm reached the 0.5 cut-off (i.e. at least 50% of service effectiveness) at baseline, with more than three-quarters of intervention facilities reaching this cut-off at endline compared to only about two-thirds of controls. About one third of control and even fewer intervention facilities reached the 0.6 cut-off at baseline. While more than half of intervention facilities eventually reached this level at endline, there was no further improvement observed in controls over time. Only single facilities (control) or none (intervention) reached the 0.7 and 0.8 cut-off scores at baseline, with slight improvements over time only in some intervention facilities. Further details on sub-scores for each of the twelve quality categories included in the composite are provided in an additional file [see Additional file 1].

**Table 2:** Obstetric care quality score and distribution of EmOC facilities by score categories

Measured quality scores	Study arm	Baseline	Midterm	Endline
Total composite score, *mean (SD)*	*Intervention*	0.55 (0.12)	0.63 (0.12)	0.65 (0.13)
	*Control*	0.56 (0.18)	0.60 (0.11)	0.53 (0.13)
Facilities with a score of 0.5 or higher, *n (%)*	*Intervention*	10 (58.8)	14 (77.8)	18 (78.3)
	*Control*	5 (55.6)	9 (75.0)	6 (66.7)
Facilities with a score of 0.6 or higher, *n (%)*	*Intervention*	3 (17.7)	7 (38.9)	13 (56.5)
	*Control*	3 (33.3)	4 (33.3)	1 (11.1)
Facilities with a score of 0.7 or higher, *n (%)*1.	*Intervention*	0 (0.0)	3 (16.7)	3 (13.0)
	*Control*	1 (11.1)	1 (8.3)	1 (11.1)
Facilities with a score of 0.8 or higher, *n (%)*	*Intervention*	0 (0.0)	0 (0.0)	0 (0.0)
	*Control*	1 (11.1)	0 (0.0)	1 (11.1)

*SD = standard deviation, 95-CI = 95%-confidence interval, n = total number, % = percentage*

Table 3 outlines the proportion of pregnant women using services at EmOC facilities in relation to the observed obstetric quality score. At baseline, most women ended up using facilities with a score of 0.59 points or less (75.4% combined in interventions areas, 62.3% combined in controls). At endline, the larger proportions of pregnant women in intervention areas used services at facilities with scores between 0.60 and 0.79 points (combined 84.1%), compared to control areas where the larger proportion of women used facilities scoring between 0.50 and 0.69 points (combined 72.6%).

**Table 3:** Women seeking care at any designated EmOC facility by obstetric care quality score (categorized)

Obstetric quality score of EmOC facility used	Study arm	Users of any EmOC service (n/*%*)		
		Baseline	Midterm	Endline
0.00—0.49 points	*Intervention*	218 (26.7)	68 (7.5)	84 (7.7)
	*Control*	131 (31.6)	55 (10.7)	38 (13.5)
0.50—0.59 points	*Intervention*	397 *(48.7)*	190 *(21.0)*	87 *(8.0)*
	*Control*	127 *(30.7)*	130 *(25.3)*	113 *(40.2)*
0.60—0.69 points	*Intervention*	194 *(23.8)*	426 *(47.1)*	519 *(47.8)*
	*Control*	58 *(14.0)*	231 *(45.0)*	91 *(32.4)*
0.70—0.79 points	*Intervention*	0 *(0.0)*	178 *(19.7)*	394 *(36.3)*
	*Control*	48 *(11.6)*	97 *(18.9)*	39 *(13.9)*
0.80—1.0 points	*Intervention*	7 *(0.9)*	42 *(4.7)*	2 *(0.2)*
	*Control*	50 *(12.1)*	0 *(0.0)*	0 *(0.0)*
Total	*Intervention*	816 *(100)*	904 *(100)*	1086 *(100)*
	*Control*	414 *(100)*	513 *(100)*	281 *(100)*

*EmOC = emergency obstetric care, 95-CI = 95%-confidence interval, n = total number, % = percentage*

### Trends in population coverage

**Fig. 1:**
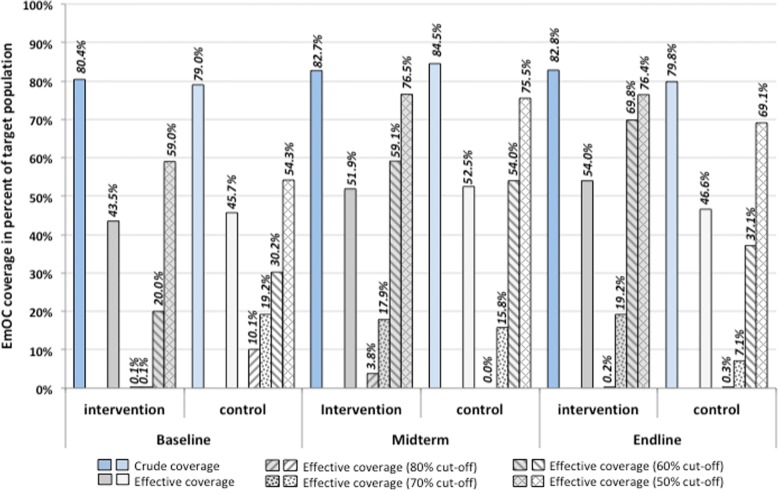
Time trends of crude and effective coverage (without and with cut-off levels applied). Data based on descriptive non-adjusted analysis

Figure 1 graphically illustrates the levels of crude and effective coverage for each study group (solid bars), as well as effective coverage for each of the different effectiveness cut-offs (patterned bars). Crude coverage remained relatively stable throughout the study period by around 80% in both study arms. In comparison, effective coverage at baseline was much lower (43.5% intervention, 45.7% control) and increased in both study arms at midterm to about 52%. At endline, effective coverage in controls declined to near baseline levels (46.6%), but remained elevated in interventions (54.0%). Applying cut-offs at different effectiveness levels, coverage trends were parallel with increases between baseline and midterm for intervention and controls at a 50% cut-off, but remained stable between midterm and endline. At a 60% cut-off, effective coverage trends were parallel between baseline and midterm and diverged between midterm and endline due to further increases in interventions and a drop in controls. At a 70% cut-off, an effective coverage upward trend between baseline and midterm occurred only in the intervention arm, with a continuous downward trend in the control arm. At an 80% cut-off, effective coverage remained extremely low for both study arms and was absent at endline.

### Effects on population coverage

Table 4 presents the effect estimate attributable to the RBF4MNH intervention modelled by our difference-in-differences analysis both at midterm (β_3_) and endline (β_6_). Effects of the RBF4MNH on crude coverage were initially negative, but slightly positive later on at endline, however, in both instances without statistical significance (*p* = 0.28 and 0.83, respectively). Effects on effective coverage in comparison were positive, but statistically non-significant, both at midterm (4.7%-points, *p* = 0.13) and at endline (7.1%-points, *p* = 0.07). At a 50% cut-off, effects on effective coverage were only minimal and statistically non-significant. At higher cut-offs, effect sizes were positive and relatively large, with a statistically significant increase of 31.1%-points (*p* = 0.02) between at endline for the 60% and of 25.8%-points (*p* = 0.04) at midterm for the 70% cut-off. At the 80% cut-off, effects remained positive, but statistically non-significant.

**Table 4:** RBF4MNH impact on crude and effective obstetric care coverage (adjusted analysis^a^)

Outcome	Estimate	Effect size (%-points)	95%-confidence interval	*p-value*
Crude coverage	β_3_ (baseline vs. midterm)	−4.3	−11.2—3.4	*0.28*
	β_5_ (baseline vs. endline)	1.2	−10.0—11.8	*0.83*
Effective coverage (no cut-off)	β_3_ (baseline vs. midterm)	4.7	−1.4—10.8	*0.13*
	β_5_ (baseline vs. endline)	7.1	−0.1—15.0	*0.07*
Effective coverage (50% cut-off)	β_3_ (baseline vs. midterm)	1.7	−22.2—25.7	*0.89*
	β_5_ (baseline vs. endline)	−1.9	−28.6—24.8	*0.89*
Effective coverage (60% cut-off)	β_3_ (baseline vs. midterm)	21.1	−8.1—50.3	*0.16*
	β_5_ (baseline vs. endline)	31.0	5.9—56.2	*0.02*
Effective coverage (70% cut-off)	β_3_ (baseline vs. midterm)	25.8	1.3—50.4	*0.04*
	β_5_ (baseline vs. endline)	16.4	−7.9—40.8	*0.19*
Effective coverage (80% cut-off)	β_3_ (baseline vs. midterm)	13.1	−8.7—43.9	*0.24*
	β_5_ (baseline vs. endline)	10.5	−10.8—31.8	*0.33*

*EmOC = emergency obstetric care, β*_*3*_ *= coefficient for effect estimate at midterm, β*_*5*_ *= coefficient for effect estimate at endline*

^a^ *Crude coverage estimates adjusted for district, distance to nearest EmOC, socioeconomic status; effective coverage estimates in addition also adjusted for facility type and ownership*

## Discussion

To date, this is the first study assessing the impact of a RBF program on effective coverage. Our findings indicate that the RBF4MNH improved effective coverage of pregnant women with facility-based obstetric care by about 7%-points after a two-year implementation period. Defining effectiveness by different quality cut-offs, our analysis further demonstrated that this impact was greatest (31%-point increase) once service effectiveness was defined as meeting at least 60% of the measured quality score. While use of obstetric care services (crude coverage) remained relatively unchanged, a higher proportion of women received higher quality services over time, although none of the studied facilities met all aspects of the quality of care measured by our score.

In LMIC contexts, RBF programs are not only seen as provider payment mechanisms but also as a driver for wider health care reforms addressing good governance, autonomy, competition, and separation of health financing functions [32]. Unlike many other RBF schemes in LMIC, which address a wide range of primary care services, the RBF4MNH kept an explicit focus on obstetric care only, and thus might not be fully comparable with other broader RBF programs. While the RBF4MNH improved quality of care (Table 1), thus allowing more women to attend services of higher quality in general (Table 3), this rather vertical implementation approach might have been less effective in addressing some of the underlying cross-cutting service delivery deficits in the country. Still, the RBF4MNH motivated health workers and management teams to take more responsibility and accountability in their strategic decision-making [33].

Given the relatively short evaluation period of about two years, the observed improvement in service quality by 0.1 points in intervention facilities is quite remarkable, but probably not sufficiently satisfying in light of the rather low scores observed at baseline (around 0.55). Although not validated, we feel confident that in developing this score using a content-of-care approach, we took sufficient precautions in both indicator selection and composite construction to adequately reflect the current standards and guidelines related to obstetric care provision in our study context beyond the programs performance focus. Thus, we consider the improvement in the score to point at wider quality of care deficits not yet sufficiently addressed by the RBF4MNH program, such as challenges related to central supply-chain management, the wider aspects related to the skilled health worker shortages, or Malawi’s declining economic situation.

Given these wider deficits in relation to health service delivery, the large positive net effects on effective coverage (20–30%-points) achieved at 60% and 70% cut-off levels are nevertheless a demonstration of the RBF4MNH’s potential to introduce improvements towards more effective service provision and coverage. However, these effects may have partly resulted from the initial RBF4MNH assignment in favour of more functional facilities. The initial upgrades (i.e. non-conditional inputs) prior to study begin might also have likely contributed to the observed effects, however, this component was considered an integrated part of the RBF4MNH implementation design and was thus evaluated accordingly. Our analytical approach, unfortunately, did not allow further discerning of the effects of upgrades from those produced by the performance payments alone.

Another reason explaining the larger effects observed at 60% and 70% effectiveness cut-offs compared to higher levels might be the relative short study period of about two years, of which the first 18 months were characterized by programmatic changes. It is therefore plausible to assume that the RBF4MNH reached full functionality only once the expansion phase started. Introduction of new purchasing structures in a healthcare setting with little antecedents in setting performance targets, service price negotiations, performance documentation and verification, or distribution and investment of rewards by providers seemingly requires a wider timeframe than the one feasible for this evaluation [34], and limited the extent to which our study was able to assess the full effect of the program.

Evidence from other RBF programs in LMIC suggests the necessity of demand-side components in redirecting women’s choice in place of delivery [9, 10]. However, we observed no substantial effects on crude coverage in this study. We attribute this to the initially relatively constrained incentives towards service use (demand-side component was only fully implemented during the expansion phase) and the fact that crude coverage was rather high already at baseline. In light of the relatively low obstetric quality observed for many facilities, a phase-in of the demand-side after successful supply-side implementation should probably have been preferential from an ethical point of view, in order to limit the extent to which women are directed to use services that provide substantially lower quality or ineffective care.

Our study has limitations. First, as already mentioned, the two-year study period might have been too short to assess the full impact of the scheme, considering the general scope of RBF schemes to not only change reimbursement structures, but concomitantly introduce a set of new management and decision-making processes. Second, in absence of randomized assignment of facilities to the RBF, our study remained limited to a quasi-experimental design. With only one observation point available prior to intervention start we failed to test the parallel trend assumption underlying the difference-in-differences method, with our effect estimates being more conservative and likely underestimating the true effect of the intervention. Third, selection of control facilities had to be limited to the four intervention districts. RBF4MNH incentives were not only provided to assigned facilities, but also to each DHMT targeting district-wide activities (e.g. supervisory visits, supply-chain management) for the benefit of all facilities in a district (including our controls). This might have contaminated our measures on service effectiveness in the control arm underestimating the overall RBF4MNH effects observed. Lastly, the content-of-care approach underlying our composite score does not account for wider aspects of healthcare quality, such as patient satisfaction or actual mortality reduction [11]. We therefore cannot make any assumptions of the RBF4MNH’s effect on elements of effectiveness other than clinical care quality.

## Conclusion

Despite these limitations, our study was able to demonstrate that the RBF4MNH program improved effective coverage of pregnant women by improving service quality and allowing a larger proportion of women to receive more effective care in the context of Malawi. We consider our findings as evidence that RBF programs have the potential to address effective coverage and thus can play a role in LMIC’s progression towards UHC. To gain further understanding on the true potential of RBF schemes on both service and population coverage, we therefore suggest a wider use of effective coverage measures in the evaluation of RBF programs, especially in LMICs.

## Additional files

Additional file 1: RBF4MNH performance indicators and quality composite score. List of RBF4MNH performance indicators; Outline of composite development; descriptive results of composite score. (PDF 468 kb)

Additional file 2: Indicator mapping. Overview of indicator mapping by indictor categories and source documents. (XLSX 34 kb)

## Abbreviations

CCT: Conditional cash transfer
DHMT: District health management team
EC: Effective coverage
EmOC: Emergency obstetric care
FBD: Facility-based deliveries
LMIC: Low- and middle-income countries
RBF: Results-based financing
RBF4MNH: Results-Based Financing For Maternal And Neonatal Health
UHC: Universal health coverage
