# Supplementary material for: Impact of results-based financing on effective obstetric care coverage: evidence from a quasi-experimental study in Malawi
Source: BMC Health Serv Res. 2018 Oct 19;18:791. doi: 10.1186/s12913-018-3589-5 (PMC6194711; doi:10.1186/s12913-018-3589-5)
Supplement: Supplementary file 1 — RBF4MNH performance indicators and quality composite score. List of RBF4MNH performance indicators; Outline of composite development; descriptive results of composite score. (PDF 468 kb) [file 12913_2018_3589_MOESM1_ESM.pdf]

Additional file 1:

RBF4MNH Performance Indicators

Table 1: RBF4MNH performance indicators for empanelled EmOC facilities**.**

1	Partograph forms completely and appropriately filled out.
2	HIV testing, and if necessary PMTCT treatment, of expectant women with unknown HIV status.
3	Women with signs of pre-eclampsia treated with Methyldopa.
4	Women with signs of eclampsia treated with Magnesium Sulphate.
5	Newborns checked at least twice daily for 48 hours after birth.
6	Percentage of newborn babies with asphyxia resuscitated during the month of reporting.
7	Maternal deaths properly audited.
8	Newborn deaths properly audited.
9	Increase in facility-based deliveries by 2.5% compared to the same quarter of the previous year (only for basic EmOC facilities with under 85% institutional delivery rates).
10	Total number of users of modern family planning methods increased by 1% compared to the previous verification period.
11	Stock cards of essential MNH medicines and commodities up to date and complete.
12	Broken maternity equipment repaired within 7 days after loss of function.
13	Water problems in maternity ward repaired within 7 days after loss of function.
14	HMIS reports submitted on time to the district health office.
15	HMIS reports are complete and accurate.
16	RBF4MNH reports submitted on time to the district health office.
17	RBF4MNH reports are complete and accurate.
18	Use of Infection Prevention and Delivery Quality Checklist (filled out and two actions taken).
19	Use of Client Exit Questionnaire (20 filled out and two actions taken).

EmOC = emergency obstetric care; HMIS = health management information system; PMTCT = Prevention of mother-to-child transmission of HIV

Table 2: RBF4MNH performance indicators for District Health Management Teams.

1	Total number of facility-based deliveries recorded across district remains stable or increases.
2	Percentage of all EmOC facilities having a minimum of one month's supply of essential maternal and newborn health medicines and commodities on day of verification.
3	Essential equipment in operating condition in all government-owned RBF-supported EmOC facilities.
4	Percentage of HMIS reports from health facilities submitted on time to district health office.
5	Percentage of HMIS reports completely and accurately submitted to the district health office.
6	Running water in all government-owned RBF-supported EmOC facilities on day of verification.
7	Functional electricity in all government-owned health facilities on day of verification.
8	All health facilities must have received supportive supervision by District Health Management Team during the quarter, with at least two actions taken by District Health Management Team.

EmOC = emergency obstetric care; HMIS = health management information system

Generation of composite score

The steps involved in deriving the composite score used to estimate effective coverage were adapted from the relevant literature [1–3] and are outlined here.

Step 1: Indicator selection

We ensured the content of both input and process indicators included in the composite to cover all relevant aspects related to the provision of obstetric care by consulting existing literature. We included four peer-reviewed articles published between 2013 and 2015 on the evaluation of quality of obstetric care in low-income settings [4–7]. We mapped the Indicators from each of these articles and grouped them into twelve quality of care categories (see Table 1 below). We matched each indicator with available data from the case observations and facility inventories. Only a few of the initially mapped indicators were not obtainable in the available datasets and thus excluded. The complete indicator map is shown in more detail [see Additional file 2].

Step 2: Indicator definition

For the majority of indicators identified the measurement definition was explicit and fully aligned with the variable definitions in our datasets. To match the definition of some of the process indicators, we had to combine several process variables and indicated this in the legend to Table 1 below. However, many of the identified input indicators related to the availability of supply or drug items did not provide sufficiently specific definitions as to the minimum number of units a facility should provide or have in stock. We therefore created two definitions for each of these indicators: one in which only one unit of the respective item was counted (relaxed definition), the other with an arbitrarily set minimum of ten units (stricter definition). All input items for which we generated a relaxed and a strict definition are highlighted in *italics* in Table 1 below. The effect of each definition type was compared in the sensitivity analysis (Step 6).

Step 3: Indicator measurements

All indicators were measured as binary variables. In aligning process (i.e. information from multiple case observations per facility) and input indicators (i.e. information from facility

inventory), we first treated each case as an individual observation and matched it with the respective inventory information. Once the weighting, rescaling, and aggregation steps were completed (see below), individual case observations were then averaged across each facility and time point to attain a single facility-specific score.

Step 4: Weighting

To account for the relative contribution of each identified process or input indicator to the overall effectiveness of obstetric care, we introduced different weights. In defining weights, we considered each of the four published articles identified in Step 1 as an individual expert opinion. Depending on how many of the four "experts" suggested the same indicator, we identified weights ranging from 1 to 4. See Table 1 below for the resulting indicator weights. Additionally, we also kept a weighting approach based on equal weights, which was later used as an alternative in the sensitivity analysis. The effect of each weighting approach was compared in the sensitivity analysis (Step 6).

Step 5: Aggregation and rescaling

We first aggregated by summing the weighted indicators for each of the twelve quality of care categories separately, resulting in twelve category scores for each observed case. As categories varied in the total number of indicators and to ensure each category contributed equally to the final composite score, we rescaled each category score to take a value between 0 and 1. To derive the final composite score, we further aggregated the category scores by adding them together and dividing them by the total number of categories.

Step 6: Sensitivity analysis

Each of the decisions taken (Steps 1-5) may have biased our composite to some degree compared to the alternative decisions forgone. We consider our composite to explicitly serve the computation of our effect measure (effective coverage) within the scope of this study, but it should not be considered a universal measure of obstetric care effectiveness applicable beyond this limited scope. Therefore, we limited our uncertainty analysis to the comparison of our "base" score (i.e. strict definition, literature derived weights) with three alternative scores (replacing strict with relaxed definition, replacing literature with equal weights,

replacing both). We assessed the stability of this base score with the three alternative scenarios at baseline (i.e. prior to expected changes) using both Pearson and Spearman rank correlation, defining strong correlation as coefficients greater than 0.70. All comparison combinations resulted in coefficients of above 0.95 and we concluded that using the base over alternative scores is unlikely to bias the resulting effective coverage measures.

Measured obstetric care quality based on composite score

Table 2 below summarizes the measured obstetric care quality scores for the facilities in our sample.

Overall, averaged total scores (range 0-1) were similar at baseline for both intervention arms. While for facilities in the intervention arm average scores increased by 0.1 points to 0.65 points from baseline to endline, scores assessed at the two follow-up time points fluctuated around the baseline score of 0.56 points.

Comparing the point scores of each of the twelve categories, we observed that in response to the standards included in our scoring approach facilities scored generally highest for the categories *initial labor status assessment* and *active management of labor* and lowest for *immediate postpartum monitoring of newborns*. Comparing trends in point scores for each category between baseline, midterm, and endline, we found strongest improvements for the categories *partograph monitoring* and *immediate postpartum monitoring of mothers* among intervention facilities and greatest declines for *EmOC* and *EmNC readiness* across facilities in both study arms.

Table 3 below presents the distribution of facilities for different score categories. About more than half of facilities in both intervention arms reached at least a score of 0.5 points at baseline. For both arms, the proportion of facilities reaching a score of at least 0.5 points increased over time. About one third of control facilities reached a score of at least 0.6 points at baseline compared to less than one fifth of intervention facilities. While the proportion of intervention facilities reaching at least a score of 0.6 points almost tripled by endline, the control proportion decreased by a factor of three. During midterm and endline few intervention facilities reached a score of at least 0.7 points, while the number of control facilities remained low at one facility throughout the study period. No intervention facility and only one control facility reached scores of 0.8 points of more.

References:

1. Nardo M, Saisana M, Saltelli A, Tarantola S. Tools for Composite Indicators Building. Ispra, Italy: European Commission Joint Research Center; 2005.

2. Jacobs R, Smith P, Goddard M. Measuring performance: An examination of composite performance indicators. Department of Health. York, UK; 2004.

3. Profit J, Kowalkowski MA, Zupancic JAF, Pietz K, Richardson P, Draper D, et al. Baby-MONITOR: A Composite Indicator of NICU Quality. Pediatrics. 2014;134:74–82.

4. Nesbitt RC, Lohela TJ, Manu A, Vesel L, Okyere E, Edmond K, et al. Quality along the Continuum: A Health Facility Assessment of Intrapartum and Postnatal Care in Ghana. PLoS ONE. 2013;8:e81089.

5. Faye A, Dumont A, Ndiaye P, Fournier P. Development of an instrument to evaluate intrapartum care quality in Senegal: evaluation quality care. Int J Qual Health Care J Int Soc Qual Health Care ISQua. 2014;26:184–9.

6. Tripathi V, Stanton C, Strobino D, Bartlett L. Development and Validation of an Index to Measure the Quality of Facility-Based Labor and Delivery Care Processes in Sub-Saharan Africa. PloS One. 2015;10:e0129491.

7. Brenner S, De Allegri M, Gabrysch S, Chinkhumba J, Sarker M, Muula AS. The quality of clinical maternal and neonatal healthcare - a strategy for identifying “routine care signal functions.” PloS One. 2015;10:e0123968.

Table 1. Categorization and weighting of process and input indicators included in quality composite score.

Quality of care categories	Categorization and weighting of process indicators		Categorization and weighting of input indicators	
**Initial vital sign assessment**	Initial BP check	3	BP machine	3
	Initial pulse check	2	Clock	2
	Initial temperature check	1	Thermometer	1
**Initial risk factor assessment**	History assessed for pre-eclampsia symptoms [a]	2	RDT HIV	1
	History assessed for vaginal bleeding episodes	2	RDT urine protein	1
	History assessed for fever or other symptoms of infection	1	RDT hemoglobin	1
	History assessed for known HIV status	1	Stethoscope	1
	Signs of pre-eclampsia checked [b]	1	RDT urine bacteria/nitrite	1
	Signs of anemia checked [c]	1		
	Signs of infection checked [d]	1		
**Initial labor assessment**	Patient asked for onset of labor	1	-- not applicable --	
	Patient asked for time of ROM	1		
	Fetal lie/position/movement checked	1		
**Partograph documentation during stage 1 labor**	Partograph used	2	Partograph forms	2
	BP documented	3	BP machine	3
	Pulse documented	2	Clock	3
	Temperature documented	2	Thermometer	2
	FHR documented	2	Fetoscope	3
	Contractions documented	2		
	Vaginal exam findings documented [e]	2		
	Fetal descent documented	1		
**Infection prevention**	Hands washed before any physical contact (exam, delivery)	2	Clean water source	3
	Sterile gloves used during vaginal exam	2	Soap	3
	Sterile gloves used during delivery	1	*Sterile gloves*	2
	Perineum cleansed before vaginal exam and delivery	1	Skin antiseptic	
	Sterile birth equipment (cord ties, blades) used	1	*Sterile delivery pack*	
**Active management stage 3 labor (AMTSL)**	Oxytocin administered	4	*Oxytocin*	4
	Controlled cord traction	3		
	Uterus palpation or massage	4		
	Completeness of placenta confirmed	3		

**Immediate newborn care**	Newborn is dried/kept warm	3	Clean towels	2
	Skin-to-skin contact	3	Newborn scale	2
	Newborn's weight is checked	2	Cord clamps/ties	1
	Newborn's responsiveness is checked	1	*Tetracycline ointment*	1
	Cord is clamped after 2-3 min	1		
	Antibiotic eye ointment is applied	1		
**Immediate postpartum monitoring mother**	BP checked at least once in 2 hours	3	BP machine	3
	Pulse checked at least once in 2 hours	3		3
	Bleeding checked at least once in 2 hours	2		
	Uterus tone checked at least once in 2 hours	2		
	Assisted initial breastfeeding within first hour	2		
**Immediate postpartum monitoring newborn**	Responsiveness checked at least once in 2 hours	2	Thermometer	1
	Temperature checked at least once in 2 hours	1		
**Patient centered-ness**	Patient offered to have a guardian with her	1	Functional patient toilet	1
	Patient oriented on birth-relevant processes and procedures	1	Functional patient washroom (water, soap)	1
**EmOC readiness**	-- not applicable --		*At least one broad-spectrum antibiotic available* [f]	1
			At least one anticonvulsant option [g]	1
			At least one isotonic fluid option [h] *plus infusion set*	1
			Vacuum extractor	1
**EmNC readiness**	Newborn resuscitation equipment set up prior to stage 2 labor	1	Newborn size bag valve and mask	2
			Dexamethasone	1
			Ability to administer Intravenous solutions (D5NS, D50, small gauge IV cannula)	1

BP = blood pressure; EmOC = emergency obstetric care; EmNC = emergency newborn care; FHR = fetal heart rate; RDT = rapid diagnostic test

[a] *includes assessment of three symptoms: headache, blurred vision, convulsion, pregnancy-induced hypertension.*

[b] *Only includes check for edema, as routine testing of urine for proteinuria rarely performed across case observations (independent of test availability at facilities).*

[c] *Only includes check of conjunctivae or palms, as routine testing of blood hemoglobin level rarely performed across case observations (independent of test availability at facilities).*

[d] *Only includes auscultation of chest, as routine testing of urine for bacteriuria rarely performed across case observations (independent of test availability at facilities).*

[e] *includes documentation of all of these three findings: degree of cervical dilation, degree of moulding, appearance of amniotic fluid.*

[f] *i.e. injectable ampicillin & gentamycin, penicillin & gentamycin, or ceftriaxone*

[g] *i.e. injectable magnesium sulphate or diazepam*

[h] *i.e. normal saline or Ringer's lactate*

Table 2. Average obstetric quality scores (mean/SD) measured by intervention arm and time point.

**Sub-scores per quality categories**	**Study arm**	**Average score** (mean/SD)		
		**Baseline**	**Midterm**	**Endline**
Initial vital sign assessment	*Intervention*	0.68 (0.35)	0.83 (0.24)	0.84 (0.25)
	Control	0.63 (0.33)	0.83 (0.24)	0.73 (0.32)
Initial risk factor assessment	*Intervention*	0.42 (0.22)	0.52 (0.23)	0.50 (0.25)
	Control	0.46 (0.26)	0.42 (0.17)	0.33 (0.15)
Initial assessment of labor status	*Intervention*	0.85 (0.23)	0.78 (0.32)	0.80 (0.30)
	Control	0.79 (0.30)	0.77 (0.29)	0.83 (0.26)
Partograph documentation of stage 1 monitoring	*Intervention*	0.33 (0.26)	0.64 (0.26)	0.60 (0.30)
	Control	0.36 (0.30)	0.64 (0.25)	0.49 (0.29)
Infection prevention measures	*Intervention*	0.52 (0.19)	0.56 (0.20)	0.57 (0.24)
	Control	0.58 (0.22)	0.62 (0.21)	0.39 (0.25)
Active management of stage 3 labor (AMTSL)	*Intervention*	0.82 (0.23)	0.78 (0.25)	0.90 (0.17)
	Control	0.79 (0.32)	0.85 (0.27)	0.73 (0.29)
Immediate newborn care	*Intervention*	0.74 (0.17)	0.85 (0.17)	0.71 (0.16)
	Control	0.64 (0.30)	0.81 (0.14)	0.76 (0.15)
Immediate postpartum monitoring of mother	*Intervention*	0.53 (0.31)	0.79 (0.23)	0.77 (0.26)
	Control	0.54 (0.33)	0.72 (0.28)	0.59 (0.33)
9. Immediate postpartum monitoring of newborn	*Intervention*	0.25 (0.22)	0.31 (0.25)	0.26 (0.38)
	Control	0.32 (0.25)	0.31 (0.22)	0.19 (0.35)
Patient centeredness	*Intervention*	0.38 (0.22)	0.56 (0.26)	0.50 (0.24)
	Control	0.54 (0.17)	0.50 (0.23)	0.50 (0.21)
EmOC readiness	*Intervention*	0.75 (0.30)	0.55 (0.30)	0.34 (0.15)
	Control	0.76 (0.23)	0.52 (0.40)	0.41 (0.29)
EmNC readiness	*Intervention*	0.62 (0.23)	0.42 (0.31)	0.66 (0.27)
	Control	0.57 (0.33)	0.32 (0.30)	0.54 (0.17)
**Total composite score**	***Intervention***	**0.55 (0.12)**	**0.63 (0.12)**	**0.65 (0.13)**
	Control	**0.56 (0.18)**	**0.60 (0.11)**	**0.53 (0.13)**
